# Development and evaluation of the gender-specific CONSTANCES job exposure matrix for physical risk factors in France

**DOI:** 10.5271/sjweh.4118

**Published:** 2023-11-01

**Authors:** Francesca Wuytack, Bradley A Evanoff, Ann Marie Dale, Fabien Gilbert, Marc Fadel, Annette Leclerc, Alexis Descatha

**Affiliations:** 1 Univ Angers, CHU Angers, Univ Rennes, Inserm, EHESP, Irset (Institut de recherche en santé, environnement et travail) - UMR_S 1085, IRSET-ESTER, SFR ICAT, CAPTV CDC, Angers, France.; 2 Division of General Medical Sciences, Washington University School of Medicine in St. Louis, St. Louis, Missouri, USA.; 3 Unité “Cohortes en Population” UMS 011 Inserm/Université de Paris/, Villejuif, France.; 4 Department of Occupational Medicine, Epidemiology and Prevention, Donald and Barbara Zucker School of Medicine, Hofstra/Northwell, USA.

**Keywords:** exposure measurement, gender, musculoskeletal disorder, physical exposure

## Abstract

**Objectives:**

This study aimed to construct and evaluate a gender-specific job exposure matrix (JEM) for 27 physical work exposures, based on self-report.

**Methods:**

We constructed a JEM using questionnaire data on current physical exposures from 29 381 male and 35 900 female asymptomatic workers aged 18–69 years in the French CONSTANCES cohort study. We excluded workers with musculoskeletal pain to reduce potential reporting bias. We grouped 27 self-reported physical exposures using the French national job codes and stratified by gender. We compared individual and group-based exposures using the performance indicators Cohen’s kappa (κ), sensitivity, specificity, and area under the receiver operating curve (AUC).

**Results:**

JEM validation showed fair-to-moderate agreement (κ 0.21–0.60) for most physical exposures for both genders except for ‘reach behind’ (poor), ‘bend neck’ (poor), ‘finger pinch‘ (poor), standing’ (good), ‘use computer screen’ (good), and ‘use keyboard or scanner’ (good). We found the highest AUC for ‘standing’ (men 0.85/ women 0.87), ‘kneel/squat’ (men 0.80/women 0.81), ‘use computer screen’ (men/women 0.81), and ‘use keyboard or scanner’ (men 0.82/ women 0.84). The AUC was <0.60 for only three exposures: ‘bend neck’ (men 0.58/women 0.57), ‘finger pinch’ (men 0.56/ women 0.55), and ‘reach behind’ (men 0.54/ women 0.51).

**Conclusion:**

The constructed JEM validation measures were comparable for men and women for all exposures. Further research will examine the predictive ability of this gender-specific JEM for musculoskeletal disorders and the relevance of gender-stratification in this process, knowing accuracy of each exposure.

The Global Burden of Disease study reports that musculoskeletal disorders (MSD) are a leading cause of disability and sick leave worldwide (1), and physical exposures at work are one of the major determinants of MSD (2–4). Job exposure matrices (JEM) have been used to estimate physical work exposures and predict the risk of MSD (5–7). A JEM is a method in occupational health research that allows estimating workers’ exposures to occupational risk factors based on job titles or occupational codes rather than individual exposure data. By estimating exposures based on job titles or occupational codes, JEM allow exposure assignment to individuals in large population cohorts where individual exposure assessment would be infeasible. Using a JEM is less expensive than collecting individual exposure data, and reduces some types of information bias compared to individual self-reported exposures. JEM can also provide exposure data when individual data collection is difficult or impossible (8, 9). While use of JEM can be a valid and efficient method to estimate work exposures in the absence of individual exposure data, they cannot capture exposure variation between workers in the same job, and thus may lead to non-differential classification of exposures, potentially reducing effect sizes compared to the use of individual exposures (6, 10). Despite these limitations, JEM can be useful in a variety of settings, including occupational health research, estimates of exposure at the population level, and compensation and surveillance efforts (11).

While most existing JEM are gender-neutral, there is increasing interest in examining differences in exposure by gender. Gender may influence the effects of occupation on health through gender differences in the content and requirements of jobs, different exposures to work factors, and gender differences in the impact of work exposures. Gender-specific (stratified by gender) JEM were recently developed in Norway (6) and Finland (5). Hanvold et al (6) concluded that gender stratification seemed important to increase the accuracy of group-based exposure estimation, since they found that the variance explained by the mechanical exposures they examined was higher among men than women. Solovieva et al (10) found that men more frequently reported high physical exposures than women when looking at individual exposure data. When examining the predictive validity of their gender-specific JEM for low-back pain, odd ratios decreased for some exposures when using group-based rather than individual self-reported exposures, with some differences seen between men and women (10). Subsequently, stratification by gender has been recommended in occupational health research (12, 13). Men and women executing the same job are not always exposed in the same way and, for some occupational groups, men and women do not report the same level of physical exposures despite having the same occupation (14). All of these factors suggest that gender-specific JEM may provide more accurate exposure assessment than JEM that do not account for gender.

This study builds on a previous JEM for physical work exposures developed in France (15). The Cohorte des consultants des Centres d’examens de santé (CONSTANCES) cohort study JEM used self-reported exposure data on physical risk factors from a preliminary sample (35 526 participants) (16). In their conclusion, the authors recommended that future work on the full CONSTANCES cohort should consider gender-specific stratification given the potential influence of gender on the frequency and magnitude of awkward postures and physical workload (17). The objective of the current study was to construct a gender-specific JEM for physical work exposures using a larger sample of the CONSTANCES cohort data and to evaluate its performance by comparing group-based exposure estimates with individual exposure estimates.

## Methods

### Population

We used data from the CONSTANCES cohort study, a large population-based study in France. We included CONSTANCES participants recruited between 2012 and 2019, aged 18–69 years, who had a job that could be coded based on their questionnaire response (www.constances.fr) (16). We only included participants who were currently working. The CONSTANCES study is restricted to individuals living in one of the CONSTANCES ‘départements’ and affiliated to the National Health Insurance Fund, which includes all salaried workers and their families, whether they are professionally active, unemployed or retired (>85% of the French population). The study population does not include agricultural and self-employed workers.

### Measures

At inclusion, data were collected using a validated questionnaire including gender, musculoskeletal pain, and job title, as well as work exposures (18, 19). Musculoskeletal pain was defined as pain in the past seven days of ≥6/10 intensity in any of the areas indicated (hand/wrist, neck, shoulder, elbow, low back, knee/leg). Several studies have found that people with musculoskeletal pain may overestimate workplace physical exposures (20–22), thus we chose to include only asymptomatic workers in the analysis. Participants with missing data on musculoskeletal pain were also excluded.

Job titles were assigned a 4-digit French *Profession et Categorie Sociale* (PCS) occupational code (23). This system includes 486 job codes that can be grouped into eight broad job categories based on the first digit of the code (of which six include current workers; the other two categories being retired people and people not currently working). To obtain reliable estimates, PCS jobs with <10 responses were grouped with comparable jobs to create adequately sized ‘occupational groups’ (a minimum of 10 valid responses for each exposure for each job group. Grouping was done independently by two experts, with disagreement resolved by consensus as previously described (15). This merging process was done separately for male and female workers. There were 280 and 352 occupational groups among female and male workers, respectively.

Physical work exposures were obtained for each job from a standardized self-reported questionnaire including 27 physical work exposures (15). We stratified data by gender and then compiled the exposures for each occupational group for men and women separately. Exposures were measured on 4- and 5-point ordinal scales except for the exposure ‘physical intensity’ which was measured on a 15-point scale. Individual ordinal exposure estimates were dichotomized using the cut-off point based on the SALTSA criteria (for evaluating the work-relatedness of upper-extremity musculoskeletal disorders), where applicable (24). For exposures with no relevant SALTSA criteria, we dichotomized as follows: a score of 1 (never/nearly never) and 2 [rarely (<2 hours/day)] were considered less exposed, and 3 [often (2–4 hours/day)] and 4 (always or nearly always) were considered exposed. In addition, we created continuous group-based exposures from the original ordinal scale using a method described in the existing gender-neutral CONSTANCES JEM by Evanoff et al (15); we converted the ordinal exposure measures to a continuous scale (time exposed in minutes): 5 minutes (rating of 1=never or nearly never), 60 minutes [rating of 2=rarely (<2 hours per day)], 180 minutes [rating of 3=often (2–4 hours per day] and 360 minutes (rating of 4=always or nearly always).

### Analyses

To construct the gender-specific JEM, we stratified the data by gender and created the exposure estimates by occupational groups for males and females. Then we calculated individual and group-based (JEM) exposure estimates. For the individual exposures, we used the dichotomized exposure variables as described above to categorize workers as exposed or not exposed. We constructed and evaluated the gender-specific JEM using methods comparable to JEM previously developed in Norway (6) and Finland (5). To calculate the occupational group-based estimates, we stratified the data by gender, and calculated group-based exposure estimates separately for men and women for each exposure within each occupational group. To define exposed and not exposed workers (ie, the JEM proportion of workers exposed) for each occupational group, we dichotomized group-based exposures as follows: if a pre-specified proportion of workers in an occupational group were exposed (based on thresholds for work-related exposure level of the individual self-report data), then the occupational group was considered exposed (score of 1), otherwise, the group was classified as non-exposed (score of 0) for that physical exposure. We computed results using four pre-specified cut off levels (20%, 30%, 40%, or 50%) and then chose the optimal cut-off level for each of the 27 exposures based on the performance indicators (sensitivity, specificity, AUC, κ). We considered all four performance indicators in deciding the optimal cut-off, but gave priority to the highest area under the receiver operating characteristics curve (AUC) and to specificity (see supplementary material, www.sjweh.fi/article/4118, appendix 1). In addition, we calculated the following measures for the group-based estimates (15): mean [standard deviation (SD)], continuous mean (SD), median (and 25^th^ and 75^th^ quartiles), bias-corrected mean, and bias-corrected continuous mean. We obtained the bias-corrected mean and bias-corrected continuous mean using empirical quantile mapping whereby mean values that were within every 1% quantile range were adjusted to reflect the respective 1% quantiles of the individual-level reported values (15, 25).

To evaluate the performance of the gender-specific JEM, we computed performance indicators separately for the male and female JEM and compared results. We used four performance indicators to compare the group-based exposure estimates with the individual exposure measures (proportion of workers exposed based on individual self-reported data) (i): Cohen’s κ coefficient to measure agreement (ii), sensitivity (proportion of exposed individuals in the individual-based estimates that are identified as exposed in the group-based estimates) (iii), specificity (proportion of less exposed individuals in the individual-based estimates that are identified as less exposed in the group-based estimates), and (iv) AUC to measure the ability of the JEM to classify exposed and non-exposed individuals. We applied the following classification for Cohen’s kappa coefficient: poor (<0.21), fair (0.21–0.40), moderate (0.41–0.60), good (0.61–0.80) and excellent (0.81–1) agreement (26). We also estimated the variance (R^2^) in individual exposure estimates that was explained by the occupational groups using analysis of variance (ANOVA). For this ANOVA, the continuous exposure measures were used as dependent variable. Statistical analyses were conducted in Stata 17 (27) and R V4.3.0 software (28) with Qmap and Haven packages.

## Results

### Description of sample

**Figure 1:**
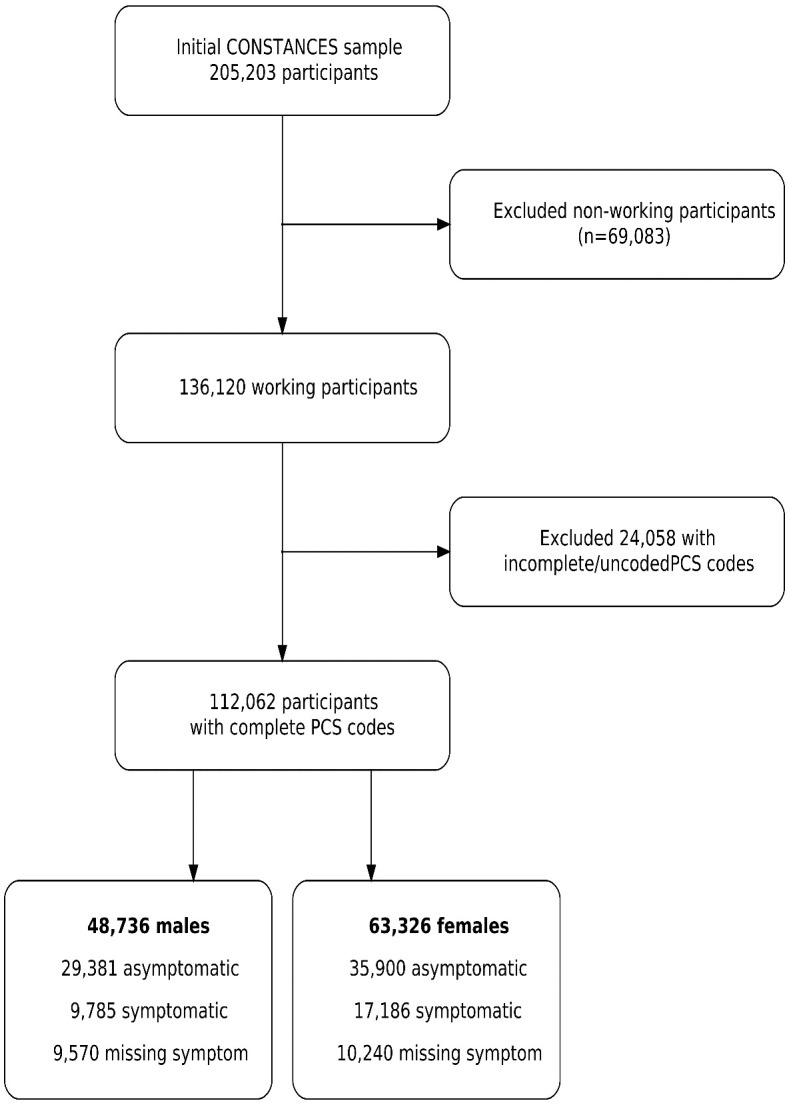
Flowchart of participants of the French CONSTANCES cohort included in the study

The CONSTANCES data set included a total of 205 203 participants. We excluded non-working participants and participants with incomplete or missing PCS codes (figure 1). Subsequently, 112 062 workers were included in the data set for analysis. After excluding workers with musculoskeletal pain, as defined above, we included 29 381 male and 35 900 female asymptomatic workers.

The mean age for men was 43.5 years (SD 10.4) and 43.2 years (SD 10.4) for women, and the mean body mass index (BMI) was 25.3 kg/m^2^ (SD 3.9) and 24.1 kg/m^2^ (SD 4.7) for men and women respectively. The most common of the six occupational categories (based on the first digit of the PCS code) for men was “executives and higher intellectual entrepreneurs” (including jobs such as doctors, engineers etc). The most common category for women was “intermediate profession” (including jobs such as schoolteachers, nurses etc) (table 1).

**Table 1:** Characteristics of working participants of the French CONSTANCES cohort with complete profession and social category codes.

Occupational category	Male (N=29 381)		Female (N=35 900)
	N (%)		N (%)
Farmers	14 (0.05)		8 (0.02)
Craftsmen, traders and entrepreneurs	598 (2.0)		225 (0.6)
Executives and higher intellectual professions	12 120 (42.3)		10 037 (28.0)
Intermediate professions	9011 (30.7)		15 894 (44.3)
Salaried employees	2776 (9.4)		8802 (24.5)

### Evaluation of the gender-specific JEM

The JEM are accessible in the supplementary material. Distribution of JEM is presented in table 2. The JEM performance measures (κ, sensitivity, specificity, AUC) using the different cut-offs (20%, 30%, 40%, 50%) to estimate the group-based exposure estimates, stratified by gender, are outlined in full in supplementary appendix 1. For most exposures the optimal cut-off was 20% or 30% (table 3). The exceptions were exposures that are common in many jobs (standing, change tasks, rest eyes, use computer screen, use keyboard or scanner), for which the optimal cut-off was 50%. The optimal cut-offs were the same for both genders.

**Table 2:** Distribution of the job exposure matrix. [SD=standard deviation, P=percentile].

		N	Mean	SD	Minimum	5^th^ P	25^th^ P	Median	75^th^ P	95^th^ P	Maximum
Men											
	Physical intensity	28 883	9.90	3.22	6	6	7	9	13	15	20
	Standing	29 012	2.58	1.06	1	1	2	2	4	4	4
	Repetition	28 585	1.65	1.02	1	1	1	1	2	4	4
	Change tasks	28 685	3.01	1.06	1	1	2	3	4	4	4
	Rest eyes	28 717	3.24	1.05	1	1	3	4	4	4	4
	Kneel or squat	29 011	1.49	0.84	1	1	1	1	2	3	4
	Bend trunk	28 962	1.54	0.88	1	1	1	1	2	4	4
	Drive machinery	29 005	1.18	0.59	1	1	1	1	1	2	4
	Drive car or truck	29 008	1.56	0.99	1	1	1	1	2	4	4
	Handle objects 1–4 kg	28 728	1.24	1.40	0	0	0	1	2	4	4
	Handle objects >4 kg	28 653	0.90	1.28	0	0	0	0	2	4	4
	Carry loads <10 kg	28 612	0.96	1.14	0	0	0	1	2	3	4
	Carry loads 10–25 kg	28 702	0.82	0.97	0	0	0	1	1	3	4
	Carry loads >25 kg	28 678	0.71	0.82	0	0	0	1	1	2	4
	Use vibrating tools	28 820	1.18	0.58	1	1	1	1	1	3	4
	Use computer screen	28 903	3.23	1.07	1	1	3	4	4	4	4
	Use keyboard or scanner	28 866	3.14	1.13	1	1	2	4	4	4	4
	Bend neck	28 815	2.19	1.06	1	1	1	2	3	4	4
	Arms above shoulder	28 891	1.33	0.67	1	1	1	1	1	3	4
	Reach behind	28 893	1.23	0.52	1	1	1	1	1	2	4
	Arms abducted	28 831	1.35	0.74	1	1	1	1	1	3	4
	Bend elbow	28 786	1.37	0.78	1	1	1	1	1	3	4
	Rotate forearm	28 845	1.29	0.69	1	1	1	1	1	3	4
	Bend wrist	28 817	1.36	0.77	1	1	1	1	1	3	4
	Press base of hand	28 837	1.17	0.53	1	1	1	1	1	2	4
	Finger pinch	28 833	1.38	0.79	1	1	1	1	1	3	4
	Work outdoors	29 176	1.51	0.90	1	1	1	1	2	4	4
Women											
	Physical intensity	35 131	9.69	3.19	6	6	7	9	12	15	20
	Standing	35 420	2.59	1.15	1	1	2	2	4	4	4
	Repetition	34 545	1.74	1.08	1	1	1	1	2	4	4
	Change tasks	34 625	2.86	1.13	1	1	2	3	4	4	4
	Rest eyes	34 592	2.98	1.17	1	1	2	3	4	4	4
	Kneel or squat	35 400	1.62	0.93	1	1	1	1	2	4	4
	Bend trunk	35 282	1.69	0.98	1	1	1	1	2	4	4
	Drive machinery	35 409	1.03	0.24	1	1	1	1	1	1	4
	Drive car or truck	35 404	1.23	0.65	1	1	1	1	1	3	4
	Handle objects 1–4 kg	34 852	1.05	1.27	0	0	0	1	2	4	4
	Handle objects >4 kg	34 751	0.70	1.13	0	0	0	0	1	3	4
	Carry loads <10 kg	34 651	0.78	1.00	0	0	0	1	1	3	4
	Carry loads 10–25 kg	34 674	0.66	0.81	0	0	0	1	1	2	4
	Carry loads >25 kg	34 705	0.61	0.74	0	0	0	1	1	2	4
	Use vibrating tools	35 015	1.04	0.29	1	1	1	1	1	1	4
	Use computer screen	35 131	3.24	1.04	1	1	3	4	4	4	4
	Use keyboard or scanner	35 073	3.15	1.11	1	1	2	4	4	4	4
	Bend neck	34 949	2.50	1.10	1	1	1	3	3	4	4
	Arms above shoulder	35 087	1.35	0.68	1	1	1	1	1	3	4
	Reach behind	35 078	1.24	0.53	1	1	1	1	1	2	4
	Arms abducted	34 992	1.30	0.70	1	1	1	1	1	3	4
	Bend elbow	34 919	1.30	0.73	1	1	1	1	1	3	4
	Rotate forearm	35 063	1.10	0.41	1	1	1	1	1	2	4
	Bend wrist	34 983	1.26	0.69	1	1	1	1	1	3	4
	Press base of hand	35 017	1.05	0.29	1	1	1	1	1	1	4
	Finger pinch	34 995	1.37	0.82	1	1	1	1	1	3	4
	Work outdoors	35 570	1.21	0.53	1	1	1	1	1	2	4

**Table 3:** Agreement measures between individual- and group-based work exposures using the optimal cut-off level by gender. [κ=kappa; AUC=area receiver operating characteristics under the curve; SENS=sensitivity; SPEC=specificity; EG=exposed group; EI=exposed individual.]

Physical exposure	Cut-off	Male asymptomatic workers									Female asymptomatic workers							
		κ	SENS (%)	SPEC (%)	AUC	EG ^a^ (%)	EI (%)	R^2 b^	R^2 c^		κ	SENS (%)	SPEC (%)	AUC	EG (%)	EI (%)	R^2 b^	R^2 c^
Physical intensity	20	0.35	72.44	82.71	0.76	22.97	10.30	0.21	0.37		0.33	64.37	85.93	0.75	18.46	8.72	0.19	0.34
Standing	50	0.70	78.43	86.27	0.85	45.25	48.71	0.51	0.58		0.74	88.50	85.16	0.87	51.13	49.27	0.61	0.64
Repetition	20	0.26	54.39	84.44	0.68	19.44	9.99	0.14	0.20		0.26	51.00	82.83	0.67	21.32	12.25	0.14	0.18
Change tasks	50	0.23	90.16	31.51	0.60	84.10	72.06	0.12	0.11		0.20	86.25	32.10	0.59	80.02	66.08	0.08	0.08
Rest eyes	50	0.36	93.07	36.28	0.66	86.44	77.43	0.19	0.20		0.35	89.25	42.91	0.66	78.96	68.00	0.20	0.21
Kneel or squat	30	0.46	71.02	84.00	0.80	23.70	14.00	0.29	0.33		0.48	81.90	79.72	0.81	31.61	18.39	0.33	0.38
Bend trunk	30	0.46	68.99	83.16	0.78	25.22	16.08	0.25	0.30		0.48	77.65	79.01	0.78	33.92	22.81	0.30	0.33
Drive machinery	20	0.41	51.12	96.21	0.72	6.12	4.94	0.30	0.35		0.20	19.03	99.52	0.59	0.62	0.76	0.09	0.10
Drive car or truck	20	0.46	68.06	86.08	0.77	22.90	16.59	0.33	0.39		0.37	49.90	94.49	0.72	8.09	5.81	0.28	0.35
Handle objects 1–4 kg	30	0.41	54.19	91.23	0.73	13.73	10.92	0.24	0.39		0.30	36.14	95.68	0.64	6.30	6.85	0.17	0.31
Handle objects >4 kg	20	0.33	65.64	88.40	0.77	15.04	6.37	0.20	0.39		0.31	49.71	94.14	0.72	7.62	4.02	0.16	0.33
Carry loads <10 kg	20	0.30	43.71	94.52	0.69	7.23	4.58	0.15	0.33		0.25	37.95	95.91	0.67	5.07	2.90	0.10	0.24
Carry loads 10–25 kg	20	0.27	36.39	97.14	0.67	3.68	2.43	0.12	0.30		0.19	28.02	97.98	0.63	2.37	1.34	0.09	0.23
Carry loads >25 kg	20	0.23	25.25	98.83	0.62	1.51	1.41	0.10	0.24		0.29	47.04	98.27	0.73	2.20	1.02	0.12	0.28
Use vibrating tools	20	0.47	71.40	90.56	0.78	16.19	10.91	0.38	0.30		0.27	32.52	97.47	0.65	3.37	2.81	0.17	0.17
Use a computer screen	50	0.60	91.52	69.17	0.80	77.14	76.30	0.47	0.47		0.62	90.47	71.79	0.81	75.29	75.61	0.47	0.46
Use keyboard or scanner	50	0.64	85.24	74.01	0.82	59.26	56.16	0.44	0.52		0.68	84.01	83.99	0.84	54.44	56.51	0.53	0.57
Bend neck	30	0.17	25.53	91.15	0.58	11.13	13.66	0.07	0.09		0.16	30.49	84.44	0.57	18.83	21.87	0.06	0.06
Arms above shoulder	20	0.28	66.47	84.79	0.71	19.17	7.72	0.19	0.26		0.26	46.11	88.80	0.67	14.03	8.10	0.13	0.18
Reach behind	20	0.11	15.17	98.56	0.54	1.90	3.40	0.07	0.09		0.05	2.72	99.83	0.51	0.27	3.67	0.03	0.04
Arms abducted	20	0.32	65.28	83.98	0.73	20.78	9.67	0.17	0.21		0.31	53.59	88.48	0.71	14.99	8.24	0.15	0.18
Bend elbow	20	0.33	66.68	83.04	0.72	22.37	10.86	0.19	0.23		0.31	47.87	89.72	0.69	13.77	9.30	0.14	0.17
Rotate forearm	20	0.45	66.84	92.97	0.75	11.74	7.87	0.34	0.38		0.22	20.27	98.81	0.60	1.63	2.31	0.11	0.14
Bend wrist	20	0.32	61.25	85.47	0.71	19.42	10.48	0.19	0.21		0.28	41.54	91.88	0.67	10.66	7.59	0.12	0.14
Press base of hand	20	0.44	64.58	90.92	0.74	15.51	11.59	0.32	0.25		0.16	14.87	98.33	0.57	2.08	3.13	0.07	0.04
Finger pinch	20	0.17	18.00	97.67	0.56	3.00	4.26	0.09	0.15		0.15	11.89	98.70	0.55	1.83	5.00	0.06	0.10
Work outdoors	20	0.46	68.89	87.37	0.77	20.94	14.77	0.34	0.42		0.31	43.49	95.52	0.70	5.97	3.82	0.19	0.27

^a^ Exposed group refers to the percentage of exposed individuals for each exposure using the group-exposure (JEM) measure estimates. ^b^ Variance in individual exposures explained by occupational group using ordinal exposure data as dependent variable. ^c^ Variance in individual exposures explained by occupational group using continuous exposure data as dependent variable.

The performance measures of the constructed gender-specific JEM were comparable for both genders across most exposures (table 3). Agreement (κ) was fair-to-moderate for most physical exposures for both genders except for the exposures of ‘reach behind’ (poor), ‘bend neck’ (poor), ‘standing’ (good), ‘use computer screen’ (good), and ‘use keyboard or scanner’ (good). We found the highest AUC for ‘standing’ (men 0.85/ women 0.87), ‘kneel/squat’ (men 0.80/women 0.81), ‘use computer screen’ (men/women 0.81), and ‘use keyboard or scanner’ (men 0.82/ women 0.84). The AUC was <0.60 for only three exposures: ‘bend neck’ (men 0.58/women 0.57), ‘finger pinch’ (men 0.56/ women 0.55), and ‘reach behind’ (men 0.54/ women 0.51).

Examining the performance of the JEM by looking at the explained variance (R^2^) is shown in table 3. The amount of variance in individual exposures explained by the occupational groups ranged from 9% (bend neck, reach behind) to 51% (standing) in men, and 3% (reach behind) to 61% (standing) in women. The largest differences in the performance measures between genders were: ‘drive machinery’ (R^2^: male 0.30; female 0.09); ‘use vibrating tools’ (R^2^: male 0.38; female 0.17); ‘rotate forearm’ (R^2^: male 0.34; female 0.11); ‘press base of hand’ (R^2^: male 0.32; female 0.07) and ‘work outdoors’ (R^2^: male 0.34, female 0.19).

## Discussion

We constructed and evaluated a gender-specific JEM for 27 physical work exposures based on self-reported data from a large national French cohort study. The gender-specific JEM showed fair-to-moderate agreement with individual self-reported physical exposure measures at work. These findings are comparable to other existing gender-specific physical exposures JEM in Finland (5) and Norway (6). JEM provide crude estimates of exposures, and it is to be expected that the accuracy of estimation varies across factors. Our JEM performed less well for the exposures reaching behind, bending neck and pinching finger. In these groups, occupational group explained little of the variation in individual exposures. For neck flexion, Hanvold et al (6) also found a lower JEM performance (AUC male 0.61; AUC female 0.62) but they did not examine reaching behind and finger pinching. Solovieva et al (5) did not examine these three exposures. We found that lower cut-offs (20%-30%) performed better for less prevalent exposures when assessing κ, sensitivity, specificity, and AUC, while a higher cut-off (50%) performed better for more prevalent exposures. This emphasizes the importance of careful selection of cut-off levels in constructing group-based exposure measures (6). To develop the JEM, we included only asymptomatic workers since symptomatic workers have been found to report higher physical exposures than asymptomatic workers in the same jobs (15, 20, 21). Also, past analyses of a JEM created from the CONSTANCES cohort showed that excluding workers with musculoskeletal symptoms created more homogenous exposure groups (16). The physical exposures in our JEM explained 4–64% of the variance in individually reported exposures. This is comparable to the Norwegian gender-specific JEM where the explained variance ranged from 7–41% (6). The wider range of explained variance in our study could be due to the larger number of physical exposures examined. Hanvold et al (6) concluded that gender-specific group-based exposure levels could be important to improve the accuracy of exposure estimates because the explained variance estimates (for the eight exposures examined) were somewhat higher among men. We found that the variance in individual exposure explained by occupational group was not always higher among men across the 27 exposures we examined, and the differences were often small. The Finnish gender-specific JEM did not examine the explained variance measure (7). These data on individual variance and group level data will also be useful in choosing exposure measures for studies of association between work exposures and MSDs.

In subsequent research, we will assess the predictive ability of this gender-specific CONSTANCES JEM for several MSD. While the agreement measures of this JEM are generally comparable for men and women, previous research has highlighted that differences can exist in physical self-reported exposures between men and women in the same occupational group (14). We will examine the impact of gender stratification when testing the predictive ability of the JEM for several MSDs. This is particularly important since women have higher years lost due to disability due to MSD than men (1). However, further steps are necessary to study the potential gain of gender stratification in terms of the predictive ability of JEM.

### Strengths and limitations

To our knowledge, this is the first gender-specific JEM of physical exposures using a large cohort (CONSTANCES) that included such a large range of physical exposures in the JEM. The 27 exposures that we measured provide a comprehensive list of physical characteristics of occupational groups. This will allow for a detailed examination of physical exposures in relation to various MSD in future studies.

Some limitations should be considered. We included a large and nationally representative study population, although the CONSTANCES sample under-represents agricultural and self-employed workers (16). We used self-reported data on physical exposures to construct the JEM, and workers might over- or underestimate exposures, leading to exposure misclassification. However, self-reported data have been shown to be useful in examining associations with MSD (29), and direct measurement is expensive and difficult to apply to large cohorts (30). We excluded workers who reported significant pain at the time of the questionnaire to reduce the potential for biased reporting of exposures due to symptoms, and because estimates based on asymptomatic workers resulted in lower within-group variance of reported exposures and created more homogeneous exposure groups in a previous JEM created using CONSTANCES data (16). Use of asymptomatic workers also reduced the mean exposures of the occupational groups, and may have reduced the representativeness of the cohort, especially with a substantial proportion as expected in such population (31, 32). However, the accuracy and reliability of the JEM’s application should be analyzed in future studies. For ease of comparison across multiple jobs and exposures, we dichotomized jobs as “exposed” or “less exposed,” as has been done in some other JEM studies. This dichotomization may have led to loss of information and could have reduced our ability to detect gender-related differences in work exposures. Finally, JEM capture an overall estimation of exposures at the time the exposure data were collected, and physical exposures in some jobs may change over time.

### Concluding remarks

We developed a gender-specific JEM for 27 physical exposures based on a large French cohort. In both genders, the JEM showed fair-to-moderate agreement with individual exposure measures. While taking into account its limitations, the JEM can provide a useful tool for exposure estimation when data on individual exposures are lacking (11, 33), and might be applied for most each exposure knowing their accuracy. The findings of the JEM validation measures were comparable for men and women for all exposures. The JEM performed less well for three exposures: reaching behind, finger pinching, and bending the neck. This suggests using caution when applying the JEM for these exposures, and the need for evaluating this ability of these JEM exposures at predicting MSD risk. JEM provide a useful tool for researchers to estimate occupational exposures among men and women in large scale epidemiological studies, especially when exposure assessment is not available. Beyond MSD outcomes, assessing biomechanical exposure on a large epidemiological scale could be useful for other research domains, including but not restricted to mental health, burden related to work in an occupational exposome approach or even work trajectories (34). Future research will examine the predictive ability of this gender-specific JEM for MSD and the relevance of gender-stratification in this process by comparing findings to a gender-neutral JEM.

### Ethical approval

The procedures followed were in accordance with the Helsinki Declaration as revised in 2008. Ethical approval was granted for CONSTANCES by the Comité d’Evaluation Ethique de l’Inserm (IRB0000388, FWA00005831), and by the CCTIRS and CNIL (15-636 and 2017-172 respectively) as part of the overarching Comett – Musculoskeletal Observatory Cohort study.

## Supplementary material

Supplementary file 1

Gender-specific CONSTANCES JEM
