# Supplementary material for: Development and evaluation of the gender-specific CONSTANCES job exposure matrix for physical risk factors in France
Source: Scand J Work Environ Health. 2023 Oct 31;49(8):549–57. doi: 10.5271/sjweh.4118 (PMC10859043; doi:10.5271/sjweh.4118)
Supplement: Supplementary file 1 [file SJWEH-49-549-S001.pdf]

Development and evaluation of the gender-specific CONSTANCES job exposure matrix for physical risk factors in France[1]

by Francesca Wuytack, PhD, Bradley A Evanoff, MD, MPH, Ann Marie Dale, PhD, Fabien Gilbert, MSc, Marc Fadel, MD, Annette Leclerc, PhD, Alexis Descatha, MD, PhD[2]

1. Supplementary material
2. Correspondence to: Alexis Descatha, INSERM U1085, ESTER team, Faculté de santé - Département Médecine, 28 rue Roger Amsler, CS 74521, 49045 Angers cedex, France. [E-mail: alexis.descatha@inserm.fr]

Appendix 1: JEM performance measures at different cut-offs to create group exposures (asymptomatic cohort)

Male cohort – cut-off 50%

Physical exposure	K	Sensitivity (%)	Specificity (%)	AUC	Exposed groups (%)	Exposed individuals (%)
Physical intensity	0.23	18.18	98.31	0.58	3.39	10.30
Standing	0.65	78.43	86.27	0.82	45.25	48.71
Repetition	0.21	16.21	98.30	0.57	3.15	9.99
Change tasks	0.25	90.16	31.51	0.61	84.10	72.06
Rest eyes	0.34	93.07	36.28	0.65	86.44	77.43
Kneel or squat	0.39	35.84	96.21	0.66	8.28	14.00
Bend trunk	0.37	38.92	93.67	0.66	11.57	16.08
Drive machinery	0.26	15.99	99.85	0.58	0.93	4.94
Drive car or truck	0.40	32.92	97.99	0.65	7.14	16.59
Handle objects 1-4 kg	0.30	24.77	97.78	0.61	4.68	10.92
Handle objects >4 kg	0.11	6.74	99.56	0.53	0.84	6.37
Carry loads <10 kg	0.01	0.31	99.97	0.50	0.04	4.58
Carry loads 10-25 kg	0.01	0.57	99.97	0.50	0.04	2.43
Carry loads >25 kg	0.50	0.50	100.00	x	0.01	1.41
Use vibrating tools	0.61	42.92	97.93	0.70	6.53	10.91
Use computer screen	0.60	91.52	69.17	0.80	77.14	76.30
Use keyboard or scanner	0.13	85.24	74.01	0.80	59.26	56.16
Bend neck	0.27	9.55	98.73	0.54	2.40	13.66
Arms above shoulder	0.00	21.16	98.43	0.60	3.08	7.72
Reach behind	0.11	0.20	100.00	0.50	0.01	3.40
Arms abducted	0.20	8.00	99.17	0.54	1.53	9.67
Bend elbow	0.45	15.00	98.57	0.57	2.90	10.86
Rotate forearm	0.23	37.65	98.48	0.68	4.37	7.87
Bend wrist	0.45	17.16	98.55	0.58	3.09	10.48
Press base of hand	0.08	38.68	97.57	0.68	6.63	11.59
Finger pinch	0.42	4.48	99.82	0.52	0.36	4.26
Work outdoors	0.23	34.73	97.95	0.66	6.88	14.77

Male cohort – cut-off 40%

Physical exposure	K	Sensitivity (%)	Specificity (%)	AUC	Exposed groups (%)	Exposed individuals (%)
Physical intensity	0.34	34.72	95.28	0.65	7.81	10.30
Standing	0.64	86.18	77.81	0.82	53.36	48.71
Repetition	0.25	21.91	97.07	0.59	4.82	9.99
Change tasks	0.10	98.39	8.91	0.54	96.35	72.06
Rest eyes	0.21	97.68	17.91	0.58	94.16	77.43
Kneel or squat	0.42	49.53	91.98	0.71	13.83	14.00
Bend trunk	0.41	55.54	88.48	0.72	18.60	16.08
Drive machinery	0.41	34.36	98.77	0.67	2.86	4.94
Drive car or truck	0.45	44.29	95.05	0.70	11.47	16.59
Handle objects 1-4 kg	0.37	35.07	96.12	0.66	7.28	10.92
Handle objects >4 kg	0.30	26.63	97.79	0.62	3.76	6.37
Carry loads <10 kg	0.13	8.70	99.44	0.54	0.94	4.58
Carry loads 10-25 kg	0.11	6.88	99.73	0.53	0.44	2.43
Carry loads >25 kg	0.01	0.74	99.98	0.50	0.03	1.41
Use vibrating tools	0.53	50.17	96.98	0.74	8.16	10.91
Use computer screen	0.57	95.48	55.97	0.76	83.29	76.30
Use keyboard or scanner	0.58	88.31	68.54	0.78	63.38	56.16
Bend neck	0.16	12.90	97.90	0.55	3.58	13.66
Arms above shoulder	0.34	30.93	97.22	0.64	4.96	7.72
Reach behind	0.05	3.26	99.78	0.52	0.32	3.40
Arms abducted	0.26	23.92	96.61	0.60	5.38	9.67
Bend elbow	0.33	33.99	95.01	0.65	8.14	10.86
Rotate forearm	0.49	46.68	97.43	0.72	6.04	7.87
Bend wrist	0.33	30.47	96.43	0.63	6.39	10.48
Press base of hand	0.49	49.93	95.46	0.73	9.80	11.59
Finger pinch	0.09	5.46	99.77	0.53	0.45	4.26
Work outdoors	0.45	41.00	96.72	0.69	8.85	14.77

Male cohort – cut-off 30%

Physical exposure	K	Sensitivity (%)	Specificity (%)	AUC	Exposed groups (%)	Exposed individuals (%)
Physical intensity	0.38	55.13	89.94	0.73	14.70	10.30
Standing	0.61	89.42	72.32	0.81	57.76	48.71
Repetition	0.29	38.85	91.93	0.65	11.15	9.99
Change tasks	0.04	99.73	3.03	0.51	98.96	72.06
Rest eyes	0.18	98.54	14.07	0.56	95.70	77.43
Kneel or squat	0.43	71.02	84.00	0.78	23.70	14.00
Bend trunk	0.42	68.99	83.16	0.76	25.22	16.08

Drive machinery	0.43	38.27	98.35	0.68	3.46	4.94
Drive car or truck	0.48	58.31	90.52	0.74	17.58	16.59
Handle objects 1-4 kg	0.41	54.19	91.23	0.73	13.73	10.92
Handle objects >4 kg	0.34	36.88	96.22	0.67	5.89	6.37
Carry loads <10 kg	0.25	21.36	98.34	0.60	2.56	4.58
Carry loads 10-25 kg	0.16	10.74	99.54	0.55	0.71	2.43
Carry loads >25 kg	0.02	0.99	99.97	0.50	0.05	1.41
Use vibrating tools	0.54	58.38	95.31	0.77	10.55	10.91
Use computer screen	0.52	97.01	47.02	0.72	86.58	76.30
Use keyboard or scanner	0.48	94.92	50.64	0.73	74.94	56.16
Bend neck	0.18	25.53	91.15	0.58	11.13	13.66
Arms above shoulder	0.35	37.43	95.78	0.67	6.78	7.72
Reach behind	0.08	4.89	99.67	0.52	0.49	3.40
Arms abducted	0.32	42.18	92.21	0.67	11.12	9.67
Bend elbow	0.36	52.48	89.58	0.71	14.99	10.86
Rotate forearm	0.51	59.67	95.23	0.77	9.09	7.87
Bend wrist	0.36	45.78	92.18	0.69	11.80	10.48
Press base of hand	0.49	54.98	94.19	0.75	11.51	11.59
Finger pinch	0.14	9.93	99.29	0.55	1.10	4.26
Work outdoors	0.50	57.29	92.48	0.75	14.87	14.77

Male cohort – cut-off 20%

Physical exposure	K	Sensitivity (%)	Specificity (%)	AUC	Exposed groups (%)	Exposed individuals (%)
Physical intensity	0.36	72.44	82.71	0.78	22.97	10.30
Standing	0.56	92.05	64.75	0.78	62.92	48.71
Repetition	0.27	54.39	84.44	0.69	19.44	9.99
Change tasks	0.03	99.83	2.41	0.51	99.20	72.06
Rest eyes	0.05	99.91	3.24	0.52	99.20	77.43
Kneel or squat	0.40	80.06	78.92	0.79	29.34	14.00
Bend trunk	0.39	76.25	77.74	0.77	30.94	16.08
Drive machinery	0.42	51.12	96.21	0.74	6.12	4.94
Drive car or truck	0.47	68.06	86.08	0.77	22.90	16.59
Handle objects 1-4 kg	0.38	73.03	83.31	0.78	22.84	10.92
Handle objects >4 kg	0.33	65.64	88.40	0.77	15.04	0.37
Carry loads <10 kg	0.30	43.71	94.52	0.69	7.23	4.58
Carry loads 10-25 kg	0.27	36.39	97.14	0.67	3.68	2.43
Carry loads >25 kg	0.23	25.25	98.83	0.62	1.51	1.41
Use vibrating tools	0.51	71.40	90.56	0.81	16.19	10.91
Use computer screen	0.41	98.55	34.15	0.66	90.80	76.30
Use keyboard or scanner	0.42	96.63	43.28	0.70	79.13	56.16
Bend neck	0.13	53.90	67.63	0.61	35.31	13.66
Arms above shoulder	0.31	66.47	84.79	0.76	19.17	7.72

Reach behind	0.17	15.17	98.56	0.57	1.90	3.40
Arms abducted	0.33	65.28	83.98	0.75	20.78	9.67
Bend elbow	0.34	66.68	83.04	0.75	22.37	10.86
Rotate forearm	0.49	66.84	92.97	0.80	11.74	7.87
Bend wrist	0.34	61.25	85.47	0.73	19.42	10.48
Press base of hand	0.48	64.58	90.92	0.78	15.51	11.59
Finger pinch	0.18	18.00	97.67	0.58	3.00	4.26
Work outdoors	0.48	68.89	87.37	0.78	20.94	14.77

Female cohort – cut-off 50%

**Physical exposure**	**K**	**Sensitivity (%)**	**Specificity (%)**	**AUC**	**Exposed groups (%)**	**Exposed individuals (%)**
Physical intensity	0.20	14.55	98.77	0.57	2.40	8.72
Standing	0.74	88.50	85.16	0.87	51.13	49.27
Repetition	0.17	12.76	98.76	0.56	2.65	12.25
Change tasks	0.20	86.25	32.10	0.59	80.02	66.08
Rest eyes	0.35	89.25	42.91	0.66	78.96	68.00
Kneel or squat	0.40	36.93	95.97	0.66	10.08	18.39
Bend trunk	0.44	51.86	89.83	0.71	19.68	22.81
Drive machinery	0.04	1.87	99.99	0.51	0.03	0.76
Drive car or truck	0.41	29.67	99.45	0.65	2.24	5.81
Handle objects 1-4 kg	0.08	5.15	99.64	0.52	0.69	6.85
Handle objects >4 kg	0.00	0.14	99.98	0.50	0.03	4.02
Carry loads <10 kg	x	x	99.99	x	0.01	2.90
Carry loads 10-25 kg	x	x	99.99	x	0.01	1.34
Carry loads >25 kg	x	x	100.00	x	0.00	1.02
Use vibrating tools	0.18	11.08	99.78	0.55	0.53	2.81
Use computer screen	0.62	90.47	71.79	0.81	75.29	75.61
Use keyboard or scanner	0.68	84.01	83.99	0.84	54.44	56.51
Bend neck	0.05	4.04	99.34	0.52	1.40	21.87
Arms above shoulder	0.08	4.68	99.75	0.52	0.61	8.10
Reach behind		x	100.00		0.00	3.67
Arms abducted	0.05	2.74	99.86	0.51	0.36	8.24
Bend elbow	0.07	4.59	99.70	0.52	0.70	9.30
Rotate forearm	0.03	1.85	99.94	0.51	0.11	2.31
Bend wrist	0.09	5.16	99.79	0.52	0.59	7.59
Press base of hand	0.05	3.19	99.83	0.52	0.27	3.13
Finger pinch	0.05	3.14	99.88	0.52	0.27	5.00
Work outdoors	0.25	16.26	99.71	0.58	0.90	3.82

Female cohort – cut-off 40%

Physical exposure	K	Sensitivity (%)	Specificity (%)	AUC	Exposed groups (%)	Exposed individuals (%)
Physical intensity	0.29	25.55	97.27	0.61	4.72	8.72
Standing	0.73	91.32	81.59	0.86	54.34	49.27
Repetition	0.23	18.90	97.67	0.58	4.36	12.25
Change tasks	0.04	99.21	4.24	0.52	98.04	66.08
Rest eyes	0.25	95.01	25.52	0.60	88.44	68.00
Kneel or squat	0.48	62.46	88.00	0.75	21.28	18.39
Bend trunk	0.49	70.58	82.99	0.77	29.23	22.81
Drive machinery	0.21	16.04	99.72	0.58	0.40	0.76
Drive car or truck	0.43	37.40	98.33	0.68	3.75	5.81
Handle objects 1-4 kg	0.16	11.55	98.83	0.55	1.88	6.85
Handle objects >4 kg	0.06	3.43	99.76	0.52	0.37	4.02
Carry loads <10 kg	0.00	0.10	99.99	0.50	0.01	2.90
Carry loads 10-25 kg	x	x	99.99	x	0.01	1.34
Carry loads >25 kg	x	x	99.99	x	0.01	1.02
Use vibrating tools	0.20	99.59	99.59	0.57	0.78	2.81
Use computer screen	0.49	96.43	45.57	0.71	86.19	75.61
Use keyboard or scanner	0.67	86.53	80.38	0.83	57.43	56.51
Bend neck	0.08	7.01	98.32	0.53	2.85	21.87
Arms above shoulder	0.12	7.57	99.47	0.54	1.10	8.10
Reach behind	0.04	2.48	99.85	0.51	0.23	3.67
Arms abducted	0.17	13.03	98.69	0.56	2.27	8.24
Bend elbow	0.21	16.33	98.24	0.57	3.12	9.30
Rotate forearm	0.12	7.05	99.78	0.53	0.38	2.31
Bend wrist	0.18	12.88	98.88	0.56	2.01	7.59
Press base of hand	0.08	5.38	99.66	0.53	0.50	3.13
Finger pinch	0.08	4.97	99.74	0.52	0.49	5.00
Work outdoors	0.27	18.32	99.53	0.59	1.15	3.82

Female cohort – cut-off 30%

Physical exposure	K	Sensitivity (%)	Specificity (%)	AUC	Exposed groups (%)	Exposed individuals (%)
Physical intensity	0.33	40.03	93.67	0.67	9.27	8.72
Standing	0.71	92.46	78.24	0.85	56.59	49.27
Repetition	0.28	29.91	94.28	0.62	8.68	12.25
Change tasks	0.02	99.70	1.95	0.51	99.14	66.08
Rest eyes	0.22	95.91	22.32	0.59	90.08	68.00
Kneel or squat	0.48	81.90	79.72	0.81	31.61	18.39
Bend trunk	0.48	77.65	79.01	0.78	33.92	22.81
Drive machinery	0.22	17.91	99.66	0.59	0.47	0.76
Drive car or truck	0.42	40.90	97.54	0.69	4.69	5.81

Handle objects 1-4 kg	0.30	33.24	95.68	0.64	6.30	6.85
Handle objects >4 kg	0.28	27.61	97.69	0.63	3.33	4.02
Carry loads <10 kg	0.12	8.37	99.38	0.54	0.84	2.90
Carry loads 10-25 kg	0.00	0.22	99.99	0.50	0.02	1.34
Carry loads >25 kg	x	x	99.99	x	0.01	1.02
Use vibrating tools	0.25	21.95	98.69	0.60	1.89	2.81
Use computer screen	0.44	97.62	38.52	0.68	88.81	75.61
Use keyboard or scanner	0.56	95.03	59.06	0.77	71.51	56.51
Bend neck	0.16	30.49	84.44	0.57	18.83	21.87
Arms above shoulder	0.19	15.17	98.06	0.57	3.02	8.10
Reach behind	0.04	2.56	99.85	0.51	0.24	3.67
Arms abducted	0.29	31.30	95.45	0.63	6.75	8.24
Bend elbow	0.31	35.37	94.31	0.65	8.45	9.30
Rotate forearm	0.19	13.72	99.42	0.57	0.88	2.31
Bend wrist	0.24	21.54	97.27	0.59	4.16	7.59
Press base of hand	0.11	8.21	99.33	0.54	0.91	3.13
Finger pinch	0.12	8.12	99.46	0.54	0.92	5.00
Work outdoors	0.32	32.74	97.64	0.65	3.52	3.82

Female cohort – cut-off 20%

Physical exposure	K	Sensitivity (%)	Specificity (%)	AUC	Exposed groups (%)	Exposed individuals (%)
Physical intensity	0.33	64.37	85.93	0.75	18.46	8.72
Standing	0.67	93.82	73.60	0.84	59.62	49.27
Repetition	0.26	51.00	82.83	0.67	21.32	12.25
Change tasks	0.01	99.95	0.79	0.50	99.70	66.08
Rest eyes	0.01	99.97	0.61	0.50	99.79	68.00
Kneel or squat	0.47	85.55	77.22	0.81	34.32	18.39
Bend trunk	0.46	83.22	74.22	0.79	38.89	22.81
Drive machinery	0.20	19.03	99.52	0.59	0.62	0.76
Drive car or truck	0.37	49.90	94.49	0.72	8.09	5.81
Handle objects 1-4 kg	0.32	62.41	88.25	0.75	15.22	6.85
Handle objects >4 kg	0.31	49.71	94.14	0.72	7.62	4.02
Carry loads <10 kg	0.25	37.95	95.91	0.67	5.07	2.90
Carry loads 10-25 kg	0.19	28.02	97.98	0.63	2.37	1.34
Carry loads >25 kg	0.29	47.04	98.27	0.73	2.20	1.02
Use vibrating tools	0.27	32.52	97.47	0.65	3.37	2.81
Use computer screen	0.32	99.25	24.48	0.62	93.47	75.61
Use keyboard or scanner	0.50	97.03	50.57	0.74	76.33	56.51
Bend neck	0.13	68.19	51.90	0.60	52.50	21.87
Arms above shoulder	0.26	46.11	88.80	0.67	14.03	8.10
Reach behind	0.05	2.72	99.83	0.51	0.27	3.67
Arms abducted	0.31	53.59	88.48	0.71	14.99	8.24

Bend elbow	0.31	47.87	89.72	0.69	13.77	9.30
Rotate forearm	0.22	20.27	98.81	0.60	1.63	2.31
Bend wrist	0.28	41.54	91.88	0.67	10.66	7.59
Press base of hand	0.16	14.87	98.33	0.57	2.08	3.13
Finger pinch	0.15	11.89	98.70	0.55	1.83	5.00
Work outdoors	0.31	43.49	95.52	0.70	5.97	3.82
